# A mixed-methods evaluation of a community physical activity program for breast cancer survivors

**DOI:** 10.1186/s12967-019-1958-4

**Published:** 2019-06-19

**Authors:** Catherine M. Sabiston, Angela J. Fong, Erin K. O’Loughlin, Sarkis Meterissian

**Affiliations:** 1 Faculty of Kinesiology and Physical Education, University of Toronto, 55 Harbord Street, Toronto, ON M5S 2W6 Canada; 2 School of Kinesiology and Health Studies, Queen’s University, Kingston, Canada; 3 Centre Hospitalier de L’université de Montréal & Individualized Program Department, Concordia University Montreal, Montreal, Canada; 4 Department of Surgery and Oncology, McGill University, Montreal, Canada

**Keywords:** Exercise, Barriers, Facilitators, Oncology, Survivorship

## Abstract

**Background:**

Given the benefits of physical activity for health and survival, clinicians are seeking opportunities for cancer patients to become more active independent of rehabilitation programs that are small, time-limited, and location specific. This proof-of-concept study evaluated a community-based physical activity program (Curves™) for increasing physical activity among women diagnosed and treated for breast cancer.

**Methods:**

Women were recruited from a breast cancer clinic through physician chart review. In study 1, women (*n *= 14) received the community physical activity memberships (Curves™), guidelines, and a pedometer. This group was compared to women (*n *= 16) who received physical activity guidelines and a pedometer on changes in physical activity. In study 2, women (*n *= 66) completed self-report questionnaires after Curves™ memberships expired to evaluate the program. Study 3 was a qualitative study exploring the benefits and barriers of the physical activity program among women (*n *= 6) who attended Curves™ regularly.

**Results:**

Provision of memberships to a community-based physical activity program did not improve physical activity levels beyond educational and information resources. However, there are a number of advantages to community-based physical activity programs, and the women offer a number of suggestions for improvements for community physical activity opportunities aimed at breast cancer survivors.

**Conclusions:**

Women-only community-based physical activity programs may be a viable option to help introduce women to get active after treatment.

*Trial registration* ISRCTN, ISRCTN14747810. Registered on 18 October 2017—Retrospectively registered, 10.1186/ISRCTN14747810

## Background

Breast cancer is the most common cancer diagnosis among Canadian women [1]. With 5-year survival rates approaching 90%, there are many women who are living with the long-term effects of breast cancer diagnosis and treatment [2]. Identifying modifiable and practical ways of reducing the acute and longer-term health burden of breast cancer is a public health priority. Increasing physical activity may be a cost-effective, feasible, safe, and effective way of helping women to manage the aftermath of breast cancer.

The benefits of physical activity (PA) for breast cancer survivors (BCS) are well-documented [3, 4]. Evidence from longitudinal research demonstrates a link between PA and reductions in risk for secondary cancers, recurrences, and cancer and all-cause mortality [5–7]. The evidence from randomized controlled trials suggests that PA improves physical, mental, and social health and well-being factors including cardiovascular fitness, physical function and weight management, reductions in depression and anxiety, and improvements in quality of life [3, 8]. Yet 50% to 90% of BCS are not meeting the recommended PA guidelines of 150 min per week of moderate to vigorous PA [9–11]. The low number of BCS engaging in health-enhancing PA, in spite of the well-documented benefits, is concerning and efforts are needed to increase opportunities for PA among BCS. In fact, the medical community is seeking PA opportunities for BCS patients independent of rehabilitation programs that are small, time-limited, and location specific [12, 13].

Community-based PA opportunities may be ideal for BCS [14, 15]. Community-based programs can include cancer-specific PA programs provided within local specialized centers and hospitals [15] or activity programs such as horseback riding [16], triathlon training [17], mountain climbing [18], and dragon boating [19]. These cancer-specific programs have shown improvements in cardiovascular fitness, strength, fatigue, psychosocial wellbeing, and posttraumatic growth among BCS [16–19]. While these PA programs specifically targeting BCS are deemed enjoyable and effective for many women, other cancer survivors report wanting to be physically active among women who have not been diagnosed with breast cancer. As such, these BCS may avoid targeted *cancer* PA programs and there is a need to identify and evaluate women-specific PA opportunities offered in the community.

Curves™ is a women-only circuit training facility with numerous locations across North America. This program may be particularly attractive to BCS as it is women-only and offers PA coaching support that is not in the context of cancer care [20]. This PA program has been shown to raise metabolic rates and to increase PA in overweight and sedentary women and, due to its popularity and the fact that it is community-based, this circuit-training PA program may lead to sustainable lifestyle changes in BCS [21, 22]. In fact, the American College of Sports Medicine roundtable on exercise guidelines for cancer survivors specifically identified the need to evaluate Curves™ as a PA program for cancer survivors [9].

**Fig. 1:**
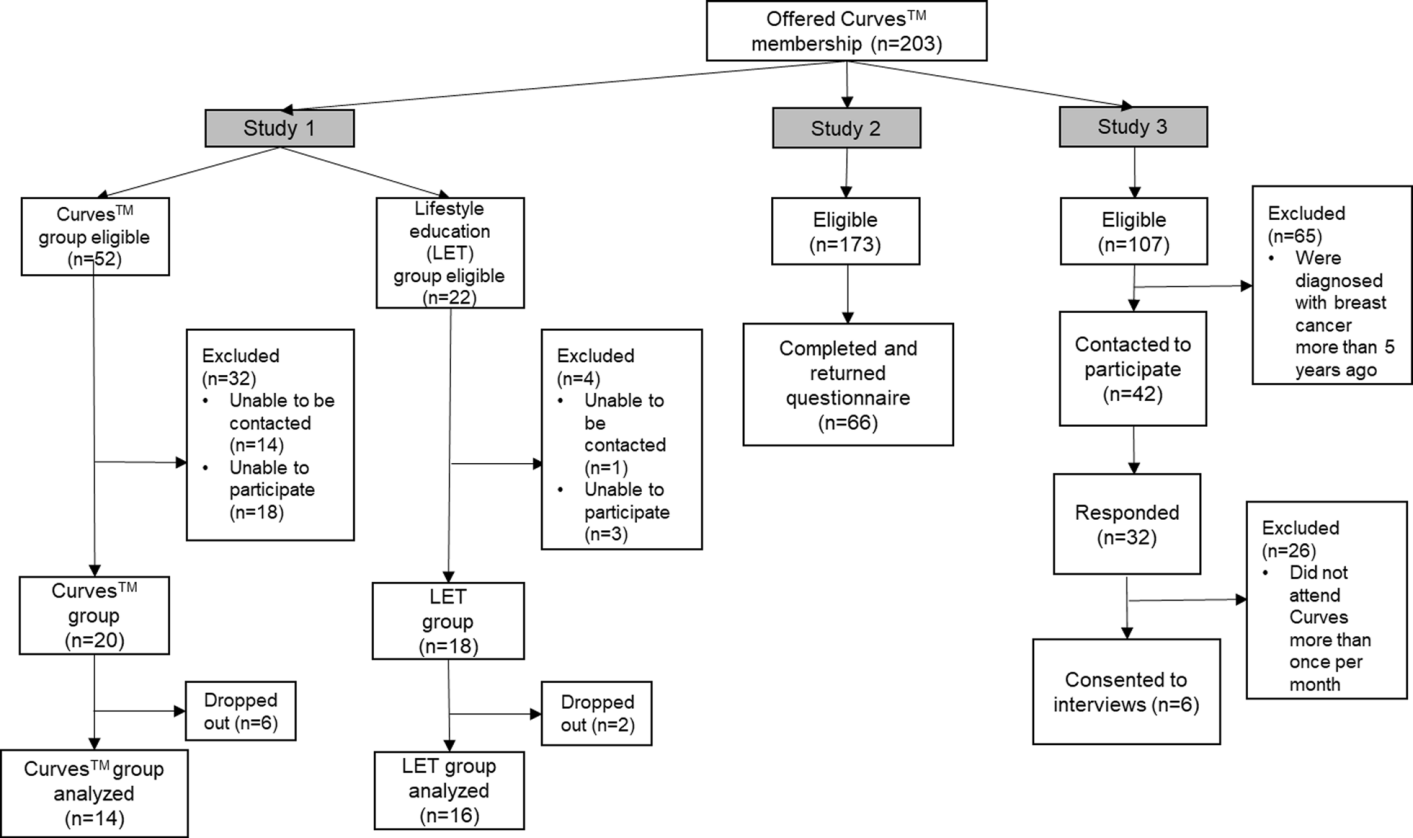
Flow of participants through all three studies. Women recruited for the Lifestyle Exercise Training (LET) group were not provided Curves™ memberships until the completion of the study. All participants across the three studies are mutually exclusive (i.e., if a participant was enrolled in one study, they are no longer eligible for the remaining two studies)

The purpose of this current proof-of-concept research was to evaluate Curves™ as a PA program for BCS. This purpose is addressed with three interrelated studies focused on a sample of BCS who were eligible for Curves™ memberships from a local breast cancer clinic in a large urban city in Canada. Specifically, 203 women accepted complimentary memberships to the PA program over a 1-year period and served as the sampling frame for the three studies in the current program of research. All participants across the three studies were mutually exclusive (i.e., only participated in one of the studies). Generally, participants were eligible for the studies if they (a) had been diagnosed with breast cancer; (b) were ≥ 18 years of age; and (c) could read and understand English or French. The flow of participants among all three studies is depicted in Fig. 1.

### Study 1

Using a proof-of-concept paradigm [23], the purpose of study 1 was to compare Curves™ and a lifestyle PA intervention on improving PA levels among BCS over 12-weeks. The lifestyle PA strategy was evidence-based [24, 25] using simple strategies such as providing PA information, guidelines and a pedometer for increasing PA behavior. Compared to structured exercise programs, lifestyle interventions have led to similar fitness gains and adherence [24, 25] and have previously demonstrated positive health benefits among BCS [26–32]. This evidence suggests that it may be feasible and efficient to target lifestyle strategies among BCS [24, 33, 34].

## Methods

### Participants and procedures

All participants were screened by the head surgical oncologist using chart review and informed of the study in person at a regularly scheduled medical appointment. Participants were randomly assigned to the community PA program (Curves™) or a lifestyle PA program. Participation in the Curves™ group involved accepting a complimentary membership in addition to general PA Guidelines [35], a cancer specific report on PA guidelines [9], and a pedometer. At the time of the study, *Exercise Guidelines for People with Cancer* were not yet published [36]. Women were asked to attend the Curves™ program as much as the PA guidelines recommend (e.g., 3 to 5 days per week) to ensure ecological validity. As an indicator of cost effectiveness, memberships were estimated at a cost of $420 per year, per person. Guidelines and materials were free, and pedometers cost the research team $12 per person.

Participants in the lifestyle PA group were given all lifestyle intervention materials without a complimentary membership. These women were asked to follow the recommendations outlined in the educational and guideline materials, which included striving towards 150 min of moderate-to-vigorous PA per week [9]. All participants completed self-report measures at baseline, were given study materials, and then completed measures 12-weeks post-baseline. Participants in the lifestyle group were offered a Curves™ membership after the 12-week intervention period.

Sample size was calculated based on the findings from Vallance et al. [25] who reported a mean difference in physical activity among BCS equating to an effect size of *d* = 0.37 and 0.38 between standard of care and either pedometer or combined print material and pedometer intervention groups. Using G*Power repeated measured analysis of variance *F*-test family with alpha = 0.05 and power of 0.08, 2 groups with 2 assessments, and a correlation among repeated measures estimated at 0.5, a total of *N* = 58 BCS were required for recruitment.

### Measures

#### Socio-demographics and cancer history

Socio-demographic variables included age; ethnicity; highest education achieved, and marital status. Cancer-related variables included stage of cancer diagnosis, time since diagnosis and treatment, and types of cancer treatment received (e.g., lymph node dissection, lumpectomy, mastectomy/double mastectomy, chemotherapy, radiotherapy, hormone therapy). Finally, body mass index (BMI) was calculated from self-reported weight and height.

#### Physical activity

Women self-reported PA using Short Questionnaire to Assess Health-Enhancing PA (SQUASH) [37] which is designed to assess daily PA during leisure time, work, school, daily transportation or other daily activities. The SQUASH has been validated against accelerometer data, which resulted in spearman correlation for overall reproducibility being 0.58 (95%-CI 0.36–0.74; [37]). Participants were asked to report daily activities by indicating the frequency, duration, and intensity of each bout. PA scores were calculated by multiplying the number of bouts per week by the number of minutes per bout, and multiplying that score by the intensity score, for each of the specific activities [36]. For the purpose of this study, only PA that could be modified (leisure, sport, and household) was totalled as the primary endpoint and is presented in Metabolic Equivalent (MET) units of energy costs [37]. To provide context, one MET is equivalent to sitting at rest, light intensity activities are ascribed a MET value around 3, moderate intensity activities are based on a MET value of around 5, and vigorous/strenuous activity is ascribed a MET value approximating 9 [37].

Women were also given a pedometer (StepsCount, Ontario, Canada) to wear each day from time awakening until bedtime except for during water activities and showering/bathing. A logbook was also provided to keep track of daily steps. Average number of steps per week was computed. For pedometer assessment to align with main analyses, average steps for week 1 (baseline) and week 12 were used in analyses.

### Data analysis

Descriptive statistics (means and standard deviations or frequencies) were calculated. To address the main purpose, a repeated measures univariate analysis of variance (RM ANOVA) model was estimated to assess differences in the PA program and lifestyle groups on PA over time. Where appropriate, Cohen’s *d* effect sizes were also calculated and reported.

## Results

A total of 38 BCS were recruited and consented to the study, although 8 women dropped out of the study before baseline assessment. A summary of demographic characteristics of the analytical sample of participants (*N *= 30; *n *= 14 Curves™ and *n *= 16 lifestyle PA) can be found in Table 1. Of note, the women in the groups were not significantly (*p *<* 0*.05) different on any measured descriptive variable. Means and standard deviations for PA levels by groups over the 12 weeks are presented in Table 2. Effect sizes are also reported. There were no significant differences in self-reported baseline PA across the groups. In the RM ANOVA, there were no significant main effects, Time *F*(1,28) = 0.80, *p *= 0.38; Group *F*(1,28) = 0.91, *p *= 0.35, or interaction, *F*(2,56) = 0.10, *p *= 0.92.

**Table 1:** Participant demographic characteristics for study 1 and study 2

Characteristics	Study 1 (n = 30)	Study 2 (n = 66)
Age (*M*(SD); range in years)	55.7 (8.9); 33 to 74	59.1 (10.9); 56 to 90
Ethnicity (% Caucasian)	87	80
Married or living with partner (%)	53	53
Children (% yes)	80	84
Highest level of education completed (% ≤ university)	33.3	29.2
Menopause (% yes)	60	63
Health and cancer-related characteristics		
Current smoker (% yes)	13.3	9.2
Weight (*M*(SD); range in kg)	70.79 (13.54); 46.7 to 99.8	74.21 (18.39); 44.6 to 134.1
BMI (kg/m^2^; *M*(SD))	26.5 (5.2)	27.9 (6.7)
Breast cancer stage diagnosis (% Stage ≤ II)	66.7	74.2
Breast cancer treatment (%)		
Lumpectomy	57	64
Single or double mastectomy	43	63
Reconstructive surgery	13	20
Chemotherapy	57	74
Radiotherapy	70	82
Hormonal therapy	63	67
Years since diagnosis (*M*(SD))	4.2 (2.8)	5.8 (3.8)

All study samples were independent and no one participant was involved in more than one study. Participant demographic characteristics for study 3 are reported in the associated results section

**Table 2:** Means and standard deviations for PA levels measured by self-report (METS) and pedometer (Steps) for the PA program and lifestyle groups in study 1

	Group	METS^a^ mean (SD)	Effect size (*d*)	Steps^b^ mean (SD)	Effect size (*d*)
Baseline	PA program	3663 (3064)	0.14	7326 (2343)	0.71
	Lifestyle	3026 (2182)		5375 (3133)	
12-weeks	PA program	3306 (2160)	0.23	6335 (1370)	0.28
	Lifestyle	2578 (1740)		5674 (3069)	

^a^METS = metabolic equivalents for physical activity

^b^Steps are per day on average over the week, rounded to closest step. For step counts, there were 8 women in the PA program group and 11 women in the lifestyle group who complied with wearing the pedometer and who are included in the analysis

For pedometer data, seven women from the Curves™ group and 12 women from lifestyle group complied with wearing the pedometer (50% and 75% compliance rates, respectively). These 19 women provided 1593 days of pedometer data and median days worn per week was seven. Means and standard deviations for the average step count over the week of assessment for the pedometer data for those who wore the device are reported in Table 2. There was no time effect, *F*(1,18) = 0.67, *p *= 0.24, group effect, *F*(1, 18) = 0.31, *p *= 0.59, or time × group interaction, *F*(2,34) = 0.93, *p *= 0.35.

### Brief summary

Overall, there were no differences in the groups based on PA participation. In this proof-of-concept study, the lack of differences may be attributed to a small sample size, additional sociocultural factors that were not measured in this study that may have influenced the groups unequally, or ineffective memberships to Curves. Understanding participation trends and experiences in the community-based program is important to better explain these findings.

### Study 2

Given the findings of the first study, the purpose of study 2 was to explore attendance at the Curves™ PA program, identify potential personal and cancer-specific factors associated with attendance, and describe the barriers and facilitators to participating in the program.

### Methods

#### Participants and procedure

One year following the provision of complimentary program memberships, 173 BCS who were not involved in study 1 were invited to participate in study 2. Sixty-six BCS (response rate of 38.2%) provided informed consent and completed a mailed questionnaire 1 year following the provision of their Curves™ membership. Questionnaires were completed and returned by mail.

### Measures

Sociodemographic and cancer history variables were assessed as described in study one.

#### Attendance

Frequency of program attendance was assessed to examine use of memberships (i.e., 1 = once; 2 = less than once a month; 3 = once per month; 4 = two to three times per month; 5 = once per week; 6 = two to three times per week; and 7 = four or more times per week). Additionally, participants were asked to describe their program participation at (i.e., 1 = I didn’t attend Curves™; 2 = I dropped out of Curves™ after trying it; 3 = I sporadically attended Curves™; and 4 = I attended Curves™ as much as I could). Curves™ PA engagement was dichotomized as either no or little participation (= 0) compared to regular participation (= 1) to further explore predictors of behavior.

#### Evaluation of the PA program

A questionnaire was developed to evaluate participants’ experiences at the Curves™ program. Questions were modeled from common theories of behavior change including Theory of Planned Behavior [38] and Expectancy-Value Model [39, 40]. Questions examined: *extent of met needs* (e.g., “To what extent did the Curves™ program meet your needs?”) rated as 1 = met none of my needs to 4 = met all of my needs, *satisfaction with Curves™ service* (e.g., Overall, how satisfied were you with the service you received at Curves™?) with responses of 1 = very dissatisfied to 4 = very satisfied, and *likelihood of choosing Curves™ in the future* (e.g., If you were to continue to exercise at a fitness facility, would you use Curves™ again?) rated as 1 = definitely not to 4 = definitely yes. Specifics of Curves™ location (distance from home, attendance patterns) were also examined.

#### Facilitators and barriers of the PA program

Women were asked to list three things they liked about the program and three things they disliked about the PA program in an open-ended question which were then coded based on common barriers for PA [41, 42] including: time, lack of social influences, energy (including fatigue), lack of motivation, injury/pain, competence and ability, and tangible factors and infrastructure. PA facilitators were coded based on common factors associated with enjoyment and PA participation [41] included: positive social influences (staff, members), variety and flexibility of opportunities, PA program specifics (e.g., duration, intensity), proximity/location, and physical and mental health outcomes.

### Data analysis

Descriptive statistics were used to characterise the sample based on sociodemographic information, cancer history and self-report PA. In the main analysis, descriptive statistics, specifically means, standard deviations and frequencies (%) were used to characterize the women’s participation (attendance) and evaluation of the Curves™ PA program. Differences in personal or cancer-related factors among women who regularly participated in the Curves™ program compared to those women who did not participate were tested using *t*-test and Chi square analyses. Open-ended responses were coded by two independent researchers and categorized into barriers and facilitators.

## Results

Participant characteristics are summarized in Table 1. The findings from study 2 are presented in Table 3. In summary, participants lived relatively close to a Curves™ location with 84% living less than 10 km away. Women attended the program 2 to 3 times per week (67%), and reported that they attended the program as much as they could (63%). However, 15% did not attend Curves™ at all and 8% dropped out after trying it once. Reasons for not attending the program were: advanced disease and on-going treatments (*n *= 5), injury precluding exercise (*n *= 1), and moved or the Curves™ gym in neighbourhood closed (*n *= 4). Reasons for drop-out, in which women could report as many as they wanted, included: poor perception of the program characteristics including loud music, preference for other activities, no health benefits attained, and distance to a PA program location.

**Table 3:** Summary of questionnaire and open-ended responses in study 2 (n = 66)

Questionnaire item	% reported
Location	
< 10 km away	84
10 km to 19 km away	11
> 20 km away	4
Self-reported attendance	
Once per month or less	24
2 to 3 times per month	3.0
2 to 3 times per week	67
4 or more times per week	6.1
Self-reported participation	
I didn’t attend Curves	15
I dropped out of Curves after trying it	7.6
I sporadically attended Curves	14.4
I attended Curves as much as I could	63
Reasons for drop-out	
Poor perception of the program characteristics (e.g., loud music, staff rapport, and ‘gossip’ among members)	44
Preference for other activities	33
No physical or mental health benefits attained	12
Distance to a physical activity program location	11
Involvement in Curves™ (“Agree” and “Highly Agree”)	% reported
Extent that the program met your needs	76
Satisfaction with service received	89
If you were to continue to exercise at a fitness facility, would you choose Curves again?	76
Favorable program characteristics	% reported
Variety and flexibility of opportunities	29
Positive social influences (staff, members)	22
Program and circuit specifics (e.g., duration, intensity, free cost)	22
Specific women-only focus	11
Quick physical and mental health outcomes	8
Proximity and location	7
Barriers to program	% reported
Lack of interest (e.g., repetitious, boring and limited exercises)	35
Distance/location and limited hours of operation	23
Lack of support from staff and negative social atmosphere	13
Poor music (too loud and genre)	11
Lack of motivation	6
Cost to continue	2
Lack of time	4

Women who attended the program were generally satisfied with their experience, reported that the program met their needs, and reported that they would choose Curves™ again if they were going to exercise at a fitness facility. However, none of the women had purchased a Curves™ membership at the time of completing the questionnaire. There were no differences between groups of women who attended Curves™ compared to those who did not on any personal or cancer-related factors.

Based on a qualitative assessment of open-ended responses to the list of facilitators and barriers pertaining to the program, there were 110 reported favourable characteristics (coded as variety and flexibility of opportunities, social influences, program/circuit specifics, women-only focus, physical and mental health outcomes, and proximity/location) and 83 barriers (lack of interest, distance/location and limited hours of operation, lack of support and negative social atmosphere, poor music, lack of motivation, cost to continue, and lack of time) of the program. See Table 3 for questionnaire and open-ended responses.

### Brief summary

There were a number of barriers and facilitators to program participation that may be used to inform future program development for community PA opportunities among women with breast cancer. Offering variety in programs and flexibility in participation, such as the “drop in” and short circuit nature of the program, appear to be critical to favourable perceptions. This finding is in line with recent empirical evidence on the importance of perceptions of variety in PA [43, 44]. The women-only focus was a valuable feature, yet the BCS reported some lack of support for fitness that may have hindered their attendance. Given that these findings were drawn from an open-ended survey, it is important to further understand the factors that are appreciated based on the Curves™ program.

### Study 3

Study 3 was a qualitative study among a unique sample of women who attended Curves™ and who were purposefully selected to participate in individual interviews aimed at understanding the experience of participating in the Curves™ PA program.

## Methods

### Participants and procedures

Women were purposefully sampled if they had attended Curves™ at least once a month for the duration of the membership and that they had been diagnosed with breast cancer within 5 years prior to study (Fig. 1). Women who participated in studies one and two were not sampled. The women identified a time and location for individual semi-structured interviews exploring their experiences of the program. Interviews lasted 45 to 60 min in length, were audio-recorded and transcribed verbatim.

### Data analysis

Audio files were transcribed verbatim and transcripts were analyzed using inductive thematic analysis [45]. Transcripts (raw data) were read multiple times by the second author and memoing was conducted to make note of developing concepts. Follow-up reading of each transcript by the second author was used to categorize similar codes into subthemes, and subthemes were organized into themes. An impartial research assistant reviewed themes for internal homogeneity to ensure fit within themes and external heterogeneity to ensure clear distinction between themes. When the author and research assistant did not agree, discussion occurred until consensus was reached. Descriptions of themes were developed, representative quotes were chosen, and all authors reviewed the themes and quotes.

## Results

Women (*N* = 6) were an average of 55 years old, identified as Caucasian (100%), mostly (67%) university educated and married (50%). They were all breast cancer survivors (100%) who had completed treatment an average of 3.7 (*SD *= 1.1) prior to the study commencing. Treatment included lumpectomy (67%), single or double mastectomy (33%), reconstructive surgery (33%), chemotherapy (50%), radiotherapy (83%) and hormonal therapy (67%). Participants reported that they attended Curves™ on a regular basis ranging from once per week to multiple times per week. Most of the women (*n *= 5) attended the PA program for almost a full year; however, none renewed their program membership. Two main themes and six sub-themes were identified generally around the motivation and barriers of the PA program (see Table 4 for quotes; pseudonyms are used for anonymity).

**Table 4:** Select quotes of themes from five breast cancer survivors in study 3

Theme	Exemplary quotes
Subtheme	
Motivational elements	
Workout atmosphere	I just really enjoyed it. The music was great. They had music on and that. And I find music makes you want to move. There wasn’t a lot of people so it wasn’t congested. (Carina) It’s a half hour, it’s intense, you continue, you don’t stop, you go to another machine…. which is very good. (Marilyn) I could only walk and that was barely so I found the circuit at [the PA program] was good. It wasn’t pushing you to the point of feeling totally unable to do it all and discouraged. But it was also giving you a variety enough that it, it made me come back. (Carina)
Goal achievement	I lost weight going to these classes. Oh, my heavens yes. The pounds kept dropping off. (Florence) Going to [the PA program] kept me normal. (Carina)
Social influence	They [trainers] were all young, and exercise oriented. They were really helpful. They gave me tips. It was them, I became quite good friends. (Florence) Staff is [sic] amazing, she was amazing. She never brought it [breast cancer] up, we never discussed it. So, it was very delicate. It was done delicately, but the caring was still there I wasn’t just another person working out. I had that little extra treatment, but [it was done] so delicately that only I knew. (Carina) And [the PA program] gives you that community feeling. That you belong to them. You know you get used to the people. (Charlotte) She [PA program staff member] called me at home when I didn’t show up for a few weeks. (Carina)
Barriers	
Location and scheduling	The problem, it was far. That was the main problem. To go with the, to take the car, to go to, that was the main [problem]. But you know to take a 15 to 20-min drive. It is a pain in the neck. Because you’re thinking you’re going to take [sic] exercise but you’re going to take the car. (Charlotte) During the summer they are closed from 11 to 1:30 or 2:30. And it doesn’t fit in with my working hours actually. (Florence) They close early in the evening. But during the summer, as soon as June 1st arrives, you have to go early in the morning because after that they’re closed till 2:30 p.m. It was just getting there and be able to rush home. (Carina)
Circuit design	It was easy. It was mentally easy, you know, I just want to go, work out and leave…. I like [the PA program] a lot, I would do it again, but I think right now for me it would be not enough. (Therese) I decided not to renew just because I started getting stronger and I started running and working more. So just the time factor you know running you can do whenever you want. In the gym, you can do whatever you want too, but it’s not quite the same, plus I needed more. (Therese) It’s boring that always it’s the same thing you know. (Charlotte)
Lack of cancer-specific support	I think that having a one-on-one with someone and finding out what their goals are and what their past experience with exercising is, is helpful. (Carina) Nobody [at the PA program] had the answers. And the answers that they were giving me I knew were wrong. (Lily) Education. Like [the PA program] bugged me because they didn’t have the knowledge. You know, it’s great to have the program with [the PA program]. Teach the people at [the PA program] the reality of breast cancer patients. You know, like…. anybody who has even one lymph node removed, is more likely to get lymphedema. Well if they’ve [the PA program staff members] never heard of lymphedema…. you know, it was like, you’re supposed to be helping me and I’m the one that knows more than you. (Lily)

Names reported are pseudonyms

### Motivational elements: “Going to Curves™ kept me normal”

Positive enablers for PA associated with Curves™ were coded as motivational elements. There were three sub-themes that developed from the data: (i) workout atmosphere, (ii) goal achievement, and (iii) social influences.

#### Workout atmosphere

Some women (33%) felt that the atmosphere created by the program was inspiring and made them feel like moving. The circuit was described as motivational based on program duration, intensity being personal and challenging yet manageable, ease of getting on machines, variety of machines, and music.

#### Goal achievement

Women mentioned weight management as a program benefit. Most (83%) also discussed the program helped them meet goals related to feeling better, finding an enjoyable activity and helped them re-gain a sense of normality.

#### Social influence

A majority (67%) of women expressed social outcomes related to attending the program. Women attending the program felt that the staff, few other BCS, and other women attending the program were supportive. Specifically, BCS felt that trainers hired at the location she attended were helpful and inspirational. They appreciated that the staff focused on them as women and not as BCS.

### Barriers: “…Plus, I needed more”

The negative or challenges to PA that were experienced or perceived at Curves™ were coded into the main theme of barriers, and there were three sub-themes: (i) location and scheduling; (ii) circuit design, and (iii) lack of cancer-specific support.

#### Location and scheduling

Lack of locations near participants’ homes was an issue. Similarly, circuit schedules and seasonal schedules were perceived as inconvenient by participants.

#### Circuit design

Two women mentioned the circuit itself did not meet their exercise intensity needs. This may have been a reason for why some women did not renew their memberships. Furthermore, half of the women mentioned a lack of program variety. While these were program challenges, it was also apparent that the program was a platform that led to other PA opportunities.

#### Lack of cancer-specific support

Improvements centred around specific types of social support. For instance, having specific, one-on-one support from staff members would be helpful for fostering attendance. Similarly, one participant felt that she lacked informational support from the program. Based on BCS’ perceptions and experiences, it was clear that further tailored social support was valuable for their interest and enjoyment.

### Brief summary

BCS who attended Curves™ experienced a number of benefits and reported a number of factors that influenced their decision to discontinue. These factors were centered on specific program logistics that could be altered for improved sustainability. Specifically, community PA programs should be accessible and flexible in hours of operation, should hire knowledgeable staff specific to breast cancer (or oncology more generally) and PA, and should offer a variety of PA plans in both number of exercises as well as intensity. Based on the strengths of the program, the duration of the activity was highly valued (e.g., 30 min). The women-only focus and motivated staff were also important. These factors overall are not surprising given the plethora of research identifying lack of perceived social support, lack of facilities or facilities that are close by and lack of motivation as barriers to engaging in PA for both BCS and other cancer survivors [14, 41, 46]. Furthermore, participants valued that staff knew they had cancer, and that there was no emphasis or focus on cancer; however, they also desired cancer and exercise-specific information.

## Discussion

The overall purpose of this research was to evaluate Curves™ as a community-based PA program for BCS. This was accomplished through three studies with independent samples, which independently aimed to (a) compare two ecologically valid programs for changes in PA levels in BCS (study 1); (b) evaluate attendance, attitudes and beliefs about characteristics of the Curves™ program (study 2); and (c) explore experiences of BCS who attended Curves™ through interviews (study 3). Based on results of study 1, Curves™ did not significantly increase PA levels in BCS who received free, 1-year memberships. Findings from studies two and three suggest that BCS who attended the program were not interested in the program for long. While a few women discussed ease of the program, there was no adaptation over time that limited interest once women improved. Nonetheless, it may be that similar community-based PA programs may be a promising opportunity for initiating PA among BCS who can then gain confidence and skill to advance to other physical activities. The women-only focus, time commitment of 30 min, simple circuit, and encouraging environment are all positive features that could be adopted in survivorship PA programs that may also be transition opportunities for BCS to gain competence, social support, and a sense of personal agency and control. In this way, sustainability of the PA program may not be a primary goal—the program can be effective at fostering a sense of normalcy among BCS and building a foundation for PA in a community setting outside of survivorship programming.

While current findings confirm there are many barriers to PA which developed from the Curves™ environment, these barriers are consistent with situational contexts of community programs more generally, including distance to closest location, hours of operation and staff [14]. Furthermore, cost of on-going memberships may have been a limitation to continued participation. Overall, providing yearly memberships to over 200 women cost an estimated $85,000. Given that Curves™ had little added impact on PA compared to a more economical intervention of education, guidelines, and a pedometer, the cost effectiveness needs to be considered. It may be that providing a free membership (that is quite costly for maintenance) is ineffective because it undermines the personal value and investment that is needed for sustainability [47]. The year-long membership may be too long of a timeframe, thus reducing the need for BCS to consistently re-evaluate in a decision process of maintaining or cancelling their commitment at the program [48]. Furthermore, from a motivation and behavior change perspective, women who are being active with free memberships because their medical team provided them with the opportunity may be a reflection of external and introjected motivation regulations that are also not likely to lead to sustainable PA [49]. As such, further consideration is needed for the appropriate model of providing community-based PA opportunities to BCS.

In contrast to previous findings, women did not discuss other common environmental or situational barriers such as lack of time, inclement weather and absence of equipment [41] or disease-related barriers such as feeling sick, fatigue and safety concerns about exercise [14, 50]. It may be that women felt supported for PA by their medical team in the act of being offered a membership and hence felt it was safe. Medical team, and primarily physician support, is a key factor in increasing PA attitudes and behaviors [51, 52]. Time and financial barriers were not likely discussed given the short circuit training model at the PA program and provision of free memberships. Furthermore, women did not discuss body image challenges that are common challenges to PA among BCS [41, 53, 54]. It may be that the women-only focus at the PA program offered a comfortable environment for PA. Given these findings, the PA program may be perceived to be a welcoming and safe environment for PA and these features are likely to enhance perceptions of competence and social support that are linked to long-term PA participation.

This work is not without its limitations. Specifically, the sample may not have been representative of BCS from this large urban centre in that they were predominately Caucasian, affluent and well-educated. The sample size for study 1 was small, and many of the women had already received memberships thus precluding random assignment to intervention groups. There are also a number of additional sociocultural factors and conditions reflecting intersections of race, identity, value systems, health perceptions, and socioeconomic position that were not assessed in study 1 that may have precluded finding differences in the intervention groups. The response rate for study 2 was low, although consistent with self-report survey research [55]. Additionally, the PA program staff or organizational leaders were not involved in the research. Future work is needed on better understanding the integration of BCS into a PA program from the program provider perspectives. In spite of these limitations, the results offer an evaluation of an understudied yet highly available PA opportunity for women.

## Conclusions

Based on strengths and weaknesses of the PA program circuit, survivorship programs can benefit from integrating women-only, supportive, small class size, short time commitment, self-paced circuit training PA features while also highlighting trained staff in oncology and fitness, adding variety and challenge, and regular adaptations based on training gains. Future interventions targeting BCS should account for these features. It is important to note that the PA program and other similar community-based programs may be a gateway opportunity to help women initiate PA in a safe and comfortable environment. Understanding the importance of community PA programs is essential for translating research into practice [56] and helping more women to increase their PA.

## Data Availability

All data generated or analyzed during this study are included in this published article. The data generated and analyzed in the current study are available from the corresponding author upon reasonable request.
